# Supplementary material for: Tuberculosis healthcare service disruptions during the COVID-19 pandemic in Brazil, India and South Africa: A model-based analysis of country-level data
Source: PLOS Glob Public Health. 2025 Jan 7;5(1):e0003309. doi: 10.1371/journal.pgph.0003309 (PMC11706508; doi:10.1371/journal.pgph.0003309)
Supplement: S1 Table — Negative values represent observed values lower than what was predicted. (DOCX) [file pgph.0003309.s002.docx]

| **TB indicator** | **Country** | **Average percent difference between observed and predicted (95% uncertainty interval)** | | |
| --- | --- | --- | --- | --- |
|  |  | **2020*** | **2021** | **2022** |
| TB tests conducted | Brazil | -24.3 (-36.6; -8.4) | -5.8 (-33.6; 42.5) | 35.8^δ^ |
|  | India | -27.8 (-34.8; -19.8) | -15.5 (-25.4; -3.7) | 44.7 (24.3; 69.9) |
|  | South Africa | -32.0 (-34.9; -28.9) | -8.3 (-12.9; -3.3) | 15.1 (5.5; 26.2)^**^ |
| Test positivity rate | Brazil | 27.4 (21.4; 33.8) | 17.8 (11.5; 24.7) | 6.3 (-0.6; 14.0) |
|  | India | 22.0 (7.1; 40.7) | 181.9 (139.3; 235.8) | 0.0 (-18.2; 24.1) |
|  | South Africa | 12.5 (-8.6; 37.4) | 5.4 ^δ^ | 9.9 ^δ **^ |
| Number of people initiating TB treatment | Brazil | -17.4 (-20.6; -13.9) | -9.4 (-13.4; -5.1) | -2.4 (-7.6; 3.2) |
|  | India | -43.3 (-46.4; -39.8) | -32.5 (-35.7; -29.2) | -33.1 (-36.4; -29.2) |
|  | South Africa | -27.0 (-36.3; -15.2) | -11.5 (-36.1; 30.2) | NA |
| Percentage successful treatment outcome | Brazil | -2.9 (-3.6; -2.2) | -3.1 (-3.8; -2.4) | NA |
|  | India | -0.04 (-0.31; 0.24) | 0.64 (0.37; 0.90) | 1.5 (1.2; 1.8) |
|  | South Africa | -4.1 (-7.1; -0.9) | NA | NA |
| Percentage unfavourable treatment outcome | Brazil | 8.1 (5.9; 10.4) | 8.4 (6.4; 10.5) | NA |
|  | India | 0.4 (-2.1; 2.9) | -6.1 (-8.4; -3.8) | -14.9 (-17.1; -12.5) |
|  | South Africa | 17.7 (3.5; 34.8) | NA | NA |
| Percentage death during treatment | Brazil | 13.7 (8.1; 19.7) | 17.6 (11.0; 24.7) | NA |
|  | India | 1.71 (-8.93; 14.0) | -11.9 (-30.7; 15.4) | -22.1 (-48.8; 30.8) |
|  | South Africa | 21.8 (7.4; 39.2) | NA | NA |
| Percentage treatment failure | Brazil | -12.6 (-32.1; 16.0) | -5.5 ^δ^ | NA |
|  | India | 29.3 (-31.7; 68.9) | 48.1 ^δ^ | 89.5 ^δ^ |
|  | South Africa | 48.75 ^δ^ | NA | NA |

^*^April 2020 -December 2020

^**^January 2022 – June 2022

NA – routine TB programmatic data was not available for this time period.

^δ^Unable to determine uncertainty interval as a log-transformation was used to prevent the ARIMA model from forecasting negative values. Logarithmic back transformations of the model prediction interval do not perfectly reflect the original data’s behavior, especially for longer forecast horizons, and so we have not reported the model uncertainty intervals here.
