# Supplementary material for: Tuberculosis healthcare service disruptions during the COVID-19 pandemic in Brazil, India and South Africa: A model-based analysis of country-level data
Source: PLOS Glob Public Health. 2025 Jan 7;5(1):e0003309. doi: 10.1371/journal.pgph.0003309 (PMC11706508; doi:10.1371/journal.pgph.0003309)
Supplement: S2 Table — The auto.arima() model used in the primary analysis is listed in the first row. Models vary by non-seasonal (p, d, q) and seasonal (P, D, Q) parameters, with [12] indicating a 12-month seasonal cycle. The table reports Akaike Information Criterion (AIC) values, Ljung–Box test p-values as well as percentage differences (with 95% uncertainty intervals) between observed and predicted values for 2020 (April–December) and 2021. (DOCX) [file pgph.0003309.s003.docx]

|  | | | | **TB indicator: Number of TB tests conducted in Brazil** | | | | | |
| --- | --- | --- | --- | --- | --- | --- | --- | --- | --- |
| **Model** | | **AIC** | **Ljung–Box test p-value (lag 5)** | Percentage difference observed vs. predicted (2020) | | | Percentage difference observed vs. predicted (2021) | | |
|  |  |  |  | **mean** | **2.5^th^ UI** | **97.5^th^ UI** | **mean** | **2.5^th^ UI** | **97.5^th^ UI** |
| Auto.ARIMA = ARIMA(0,1,1)(0,1,0)_[12]_ | | 501.54 | 0.61 | -24.3 | -36.6 | -8.4 | -5.8 | -33.6 | 42.5 |
| p=1 | ARIMA(1,1,1)(0,1,0)_[12]_ | 502.99 | 0.60 | -24.4 | -36.4 | -8.9 | -6.3 | -33.4 | 42.1 |
| p=2 | ARIMA(2,1,1)(0,1,0)_[12]_ | 503.31 | 0.98 | -22.6 | -35.0 | -6.5 | -0.3 | -32.0 | 58.9 |
| q=2 | ARIMA(0,1,2)(0,1,0)_[12]_ | 502.99 | 0.61 | -24.3 | -36.4 | -8.5 | -5.9 | -32.7 | 41.8 |
| q=0 | ARIMA(0,1,0)(0,1,0)_[12]_ | 508.66 | 0.14 | -0.9 | -54.4 | 173.8 | -262.9 | -1032.8 | 899.1 |
| P=1 | ARIMA(0,1,1)(1,1,0)_[12]_ | 502.71 | 0.45 | -25.7 | -37.3 | -10.7 | -9.6 | -34.2 | 32.0 |
| P=2 | ARIMA(0,1,1)(2,1,0)_[12]_ | 502.03 | 0.90 | -20.1 | -28.5 | -10.1 | 13.6 | -12.2 | 53.0 |
| Q=1 | ARIMA(0,1,1)(0,1,1)_[12]_ | 502.8 | 0.49 | -25.0 | -36.8 | -9.8 | -8.0 | -33.8 | 35.8 |
| Q=2 | ARIMA(0,1,1)(0,1,2)_[12]_ | 502.82 | 0.78 | -26.8 | -35.3 | -16.8 | -6.3 | -27.0 | 24.6 |
| d=0 | ARIMA(0,0,1)(0,1,0)_[12]_ | 528.44 | 0.58 | -22.7 | -33.2 | -9.6 | -4.0 | -19.2 | 16.0 |
| D=0 | ARIMA(0,1,1)(0,0,0)_[12]_ | 744.73 | 0.64 | -7.0 | -38.8 | 56.1 | 21.1 | -40.2 | 256.9 |

AIC: Akaike Information Criterion; UI: Uncertainty interval
