# Supplementary material for: Tuberculosis healthcare service disruptions during the COVID-19 pandemic in Brazil, India and South Africa: A model-based analysis of country-level data
Source: PLOS Glob Public Health. 2025 Jan 7;5(1):e0003309. doi: 10.1371/journal.pgph.0003309 (PMC11706508; doi:10.1371/journal.pgph.0003309)
Supplement: S3 Table — The auto.arima() model used in the primary analysis is listed in the first row. Models vary by non-seasonal (p, d, q) and seasonal (P, D, Q) parameters, with [12] indicating a 12-month seasonal cycle. The table reports Akaike Information Criterion (AIC) values, Ljung–Box test p-values as well as percentage differences (with 95% uncertainty intervals) between observed and predicted values for 2020 (April–December) and 2021. (DOCX) [file pgph.0003309.s004.docx]

|  | | | | **TB indicator: Number of TB tests conducted in India** | | | | | |
| --- | --- | --- | --- | --- | --- | --- | --- | --- | --- |
| **Model** | | **AIC** | **Ljung–Box test p-value (lag 5)** | Percentage difference observed vs. predicted (2020) | | | Percentage difference observed vs. predicted (2021) | | |
|  |  |  |  | **mean** | **2.5^th^ UI** | **97.5^th^ UI** | **mean** | **2.5^th^ UI** | **97.5^th^ UI** |
| Auto.ARIMA = ARIMA(0,0,0)(0,1,0)_[12]_ | | 114.54 | 0.56 | -27.9 | -34.77 | 8.0 | -15.5 | 9.9 | -25.4 |
| p=1 | ARIMA(1,0,0)(0,1,0)_[4]_ | 114.98 | 0.89 | -31.1 | -39.90 | 11.2 | -20.7 | 15.8 | -36.5 |
| p=2 | ARIMA(2,0,0)(0,1,0)_[4]_ | Non-stationary autoregressive (AR) component | | | | | | | |
| q=1 | ARIMA(0,0,1)(0,1,0)_[4]_ | 115.38 | 0.69 | -27.6 | -36.38 | 10.8 | -15.9 | 12.6 | -28.5 |
| q=2 | ARIMA(0,0,2)(0,1,0)_[4]_ | 117.38 | 0.67 | -27.7 | -36.64 | 11.1 | -15.9 | 12.8 | -28.7 |
| P=1 | ARIMA(0,0,0)(1,1,0)_[4]_ | Initial parameter values producing NaN | | | | | | | |
| P=2 | ARIMA(0,0,0)(2,1,0)_[4]_ | Initial parameter values producing NaN | | | | | | | |
| Q=1 | ARIMA(0,0,0)(0,1,1)_[4]_ | Hessian matrix is singular | | | | | | | |
| Q=2 | ARIMA(0,0,0)(0,1,2)_[4]_ | 118.54 | 0.56 | -27.8 | -34.81 | 8.2 | -15.6 | 9.8 | -25.3 |
| d=1 | ARIMA(0,1,0)(0,1,0)_[4]_ | 84.99 | 0.95 | -35.7 | -46.95 | 15.0 | -30.2 | 22.0 | -52.2 |
| D=0 | ARIMA(0,0,0)(0,0,0)_[4]_ | 219.75 | 0.55 | -25.2 | -29.49 | 4.8 | -12.3 | 4.4 | -16.6 |

AIC: Akaike Information Criterion; UI: Uncertainty interval
