# Supplementary material for: Tuberculosis healthcare service disruptions during the COVID-19 pandemic in Brazil, India and South Africa: A model-based analysis of country-level data
Source: PLOS Glob Public Health. 2025 Jan 7;5(1):e0003309. doi: 10.1371/journal.pgph.0003309 (PMC11706508; doi:10.1371/journal.pgph.0003309)
Supplement: S4 Table — The auto.arima() model used in the primary analysis is listed in the first row. Models vary by non-seasonal (p, d, q) and seasonal (P, D, Q) parameters, with [12] indicating a 12-month seasonal cycle. The table reports Akaike Information Criterion (AIC) values, Ljung–Box test p-values as well as percentage differences (with 95% uncertainty intervals) between observed and predicted values for 2020 (April–December) and 2021. (DOCX) [file pgph.0003309.s005.docx]

|  | | | | **TB indicator: Number of TB tests conducted in South Africa** | | | | | | |
| --- | --- | --- | --- | --- | --- | --- | --- | --- | --- | --- |
| **Model** | | **AIC** | **Ljung–Box test p-value (lag 5)** | Percentage difference observed vs. predicted (2020) | | | | Percentage difference observed vs. predicted (2021) | | |
|  |  |  |  | **mean** | **2.5^th^ UI** | **97.5^th^ UI** | **mean** | | **2.5^th^ UI** | **97.5^th^ UI** |
| Auto.ARIMA = ARIMA(0,0,0)(0,1,0)_[12]_ | | 565.45 | 0.34 | -32.0 | -34.9 | -28.9 | -8.3 | | -12.9 | -3.3 |
| p=1 | ARIMA(1,0,0)(0,1,0)_[12]_ | 567.33 | 0.40 | -32.0 | -35.0 | -28.8 | -8.3 | | -13.1 | -3.3 |
| p=2 | ARIMA(2,0,0)(0,1,0)_[12]_ | 569.21 | 0.39 | -32.0 | -35.2 | -28.5 | -8.3 | | -13.4 | -2.8 |
| q=1 | ARIMA(0,0,1)(0,1,0)_[12]_ | 567.34 | 0.39 | -32.0 | -35.0 | -28.8 | -8.3 | | -13.0 | -3.1 |
| q=2 | ARIMA(0,0,2)(0,1,0)_[12]_ | 569.22 | 0.35 | -32.0 | -35.2 | -28.7 | -8.3 | | -13.1 | -2.9 |
| P=1 | ARIMA(0,0,0)(1,1,0)_[12]_ | 565.9 | 0.15 | -32.7 | -35.3 | -29.8 | -8.5 | | -12.2 | -4.7 |
| P=2 | ARIMA(0,0,0)(2,1,0)_[12]_ | Poor model fit | | | | | | | | |
| Q=1 | ARIMA(0,0,0)(0,1,1)_[12]_ | 565.65 | 0.13 | -32.4 | -35.0 | -29.7 | -8.6 | | -12.0 | -5.0 |
| Q=2 | ARIMA(0,0,0)(0,1,2)_[12]_ | 567.34 | 0.17 | -32.2 | -34.4 | -29.8 | -8.8 | | -11.6 | -6.0 |
| d=1 | ARIMA(0,1,0)(0,1,0)_[12]_ | 560.16 | 0.01 | -31.2 | -52.1 | 7.4 | 21.2 | | -57.3 | 346.6 |
| D=0 | ARIMA(0,0,0)(0,0,0)_[12]_ | 886.56 | 0.19 | -32.0 | -38.0 | -25.0 | -8.2 | | -15.3 | -0.2 |

AIC: Akaike Information Criterion; UI: Uncertainty interval
