# Supplementary figures and images for: Tuberculosis healthcare service disruptions during the COVID-19 pandemic in Brazil, India and South Africa: A model-based analysis of country-level data
Source: PLOS Glob Public Health. 2025 Jan 7;5(1):e0003309. doi: 10.1371/journal.pgph.0003309 (PMC11706508; doi:10.1371/journal.pgph.0003309)

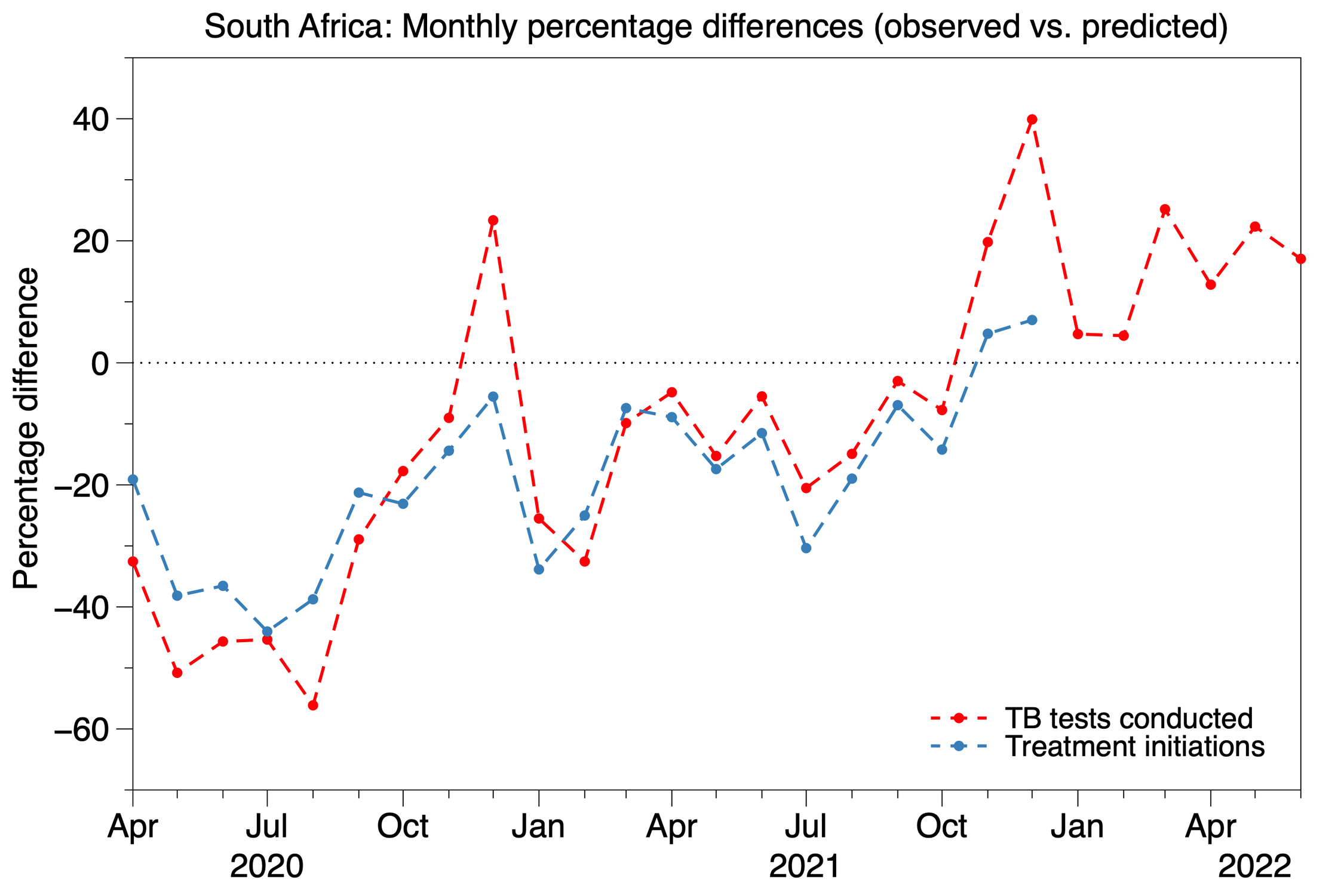

Supplement: S1 Fig — Negative values represent observed values lower than what was predicted. (TIF) [file pgph.0003309.s008.tif]

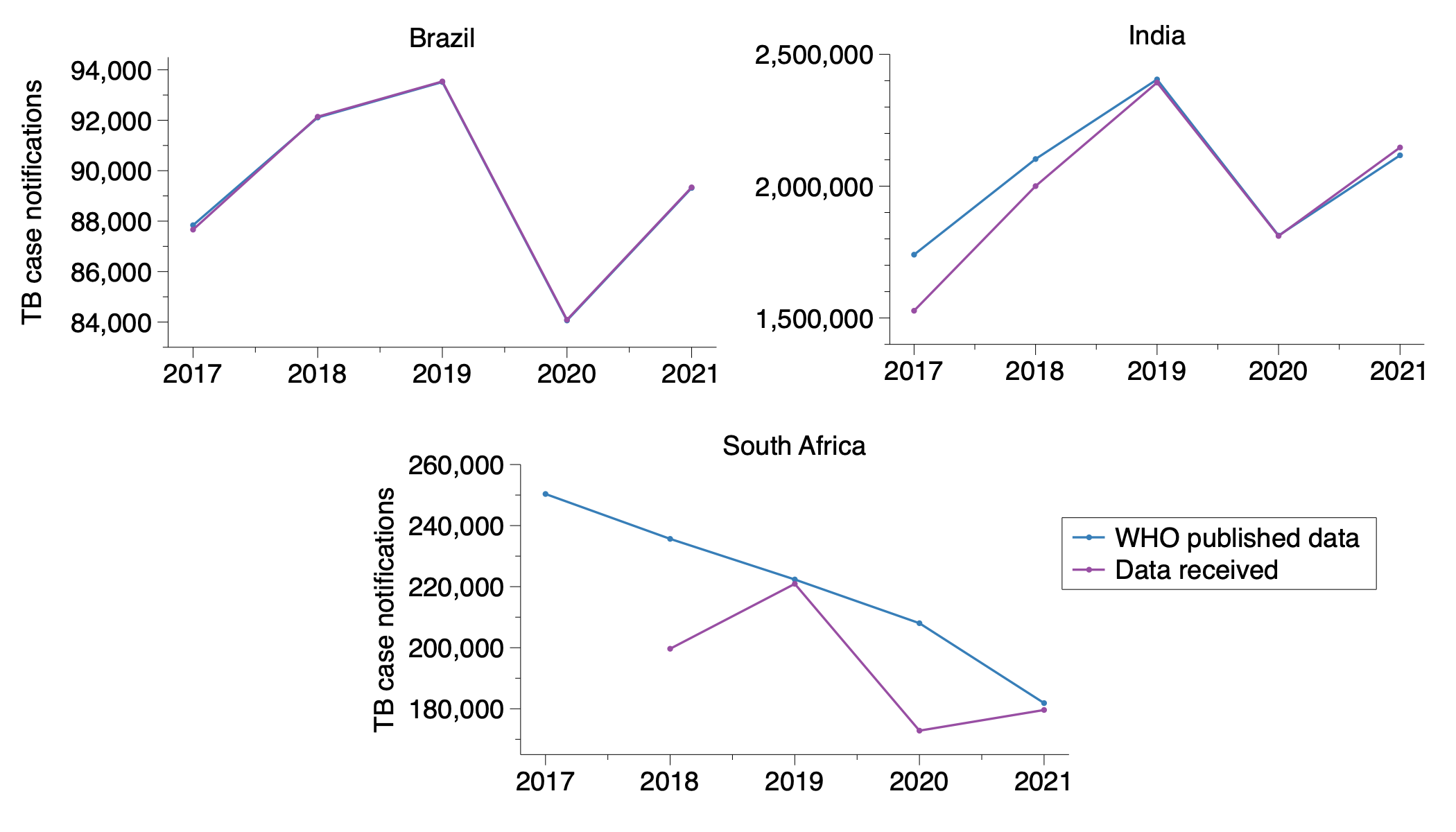

Supplement: S2 Fig — Blue lines represent WHO published data and the purple lines show the TB data we received for this study from the relevant TB programmes. WHO data was extracted from the 2022 Global Tuberculosis report. (TIF) [file pgph.0003309.s009.tif]

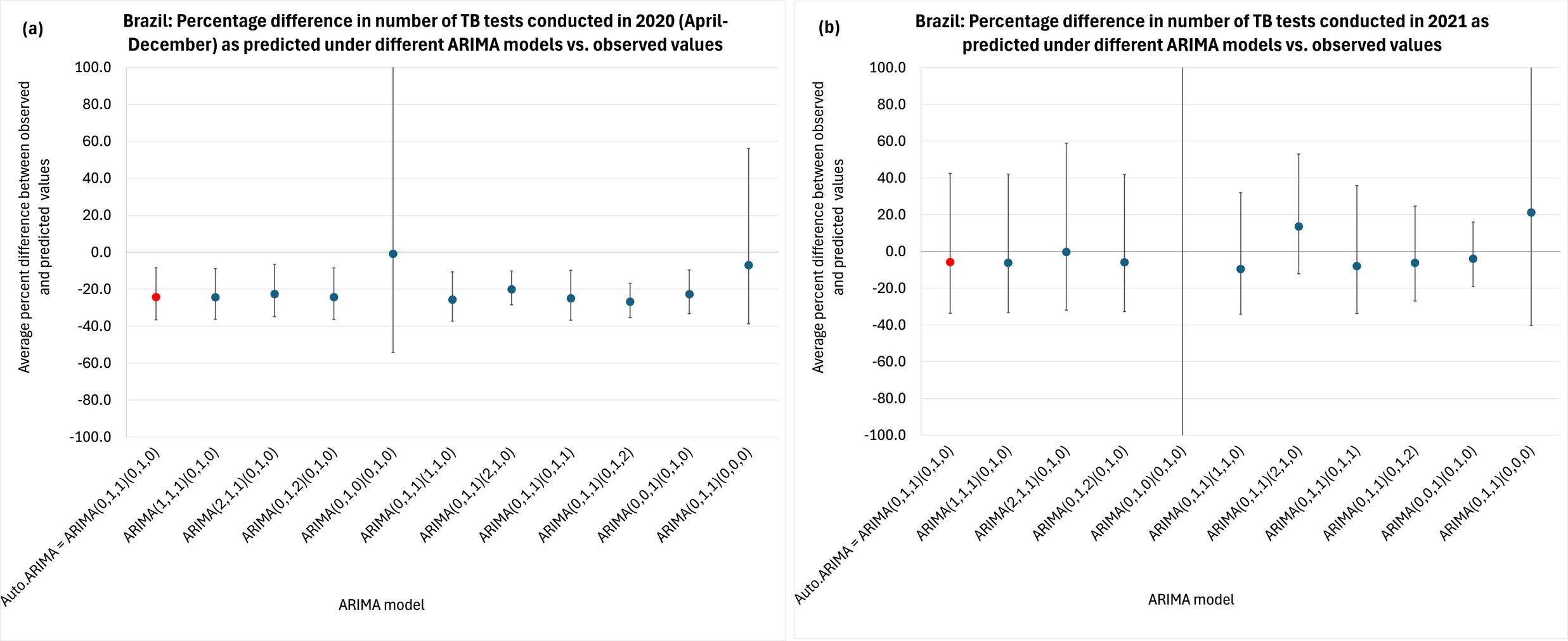

Supplement: S3 Fig — Panel (a) shows percentage differences for 2020 (April–December), and panel (b) for 2021. The Auto.ARIMA model used in the primary analysis is highlighted in red. Error bars represent uncertainty intervals for each model’s prediction. (TIF) [file pgph.0003309.s010.tif]

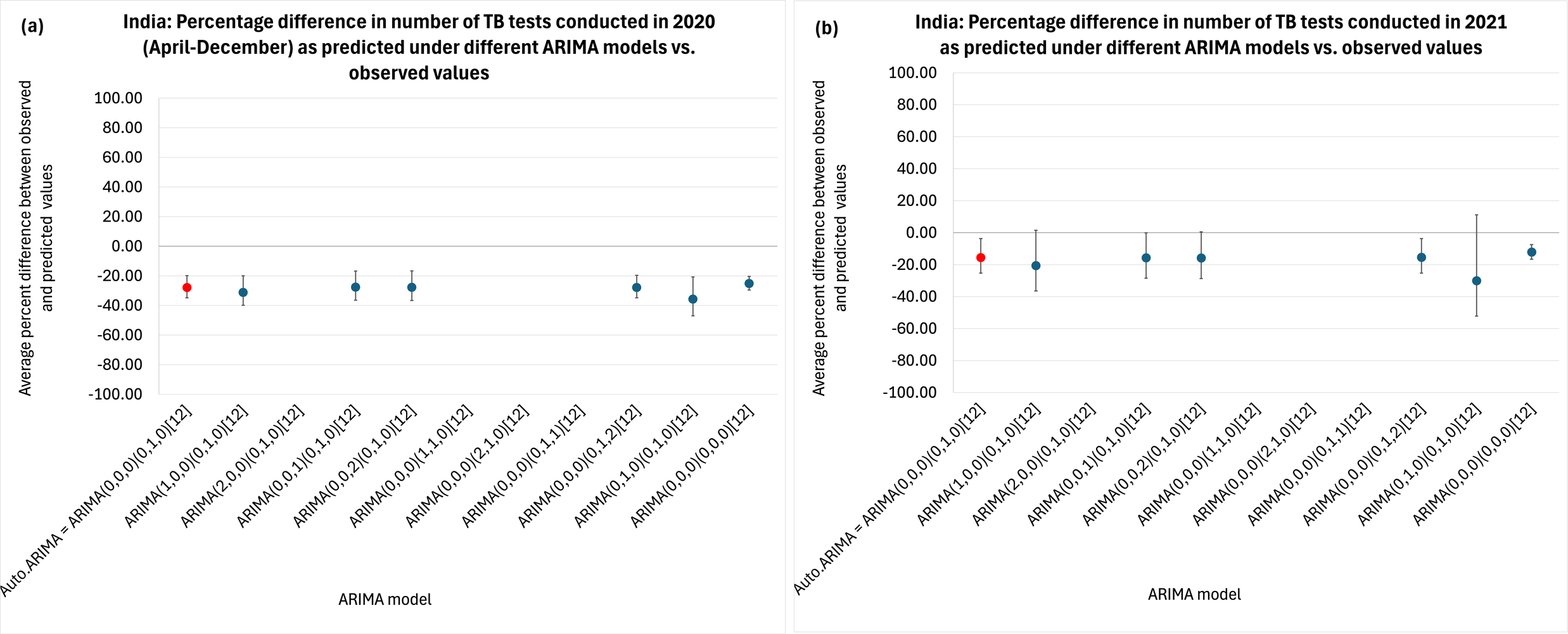

Supplement: S4 Fig — Panel (a) shows percentage differences for 2020 (April–December), and panel (b) for 2021. The Auto.ARIMA model used in the primary analysis is highlighted in red. Error bars represent uncertainty intervals for each model’s prediction. (TIF) [file pgph.0003309.s011.tif]

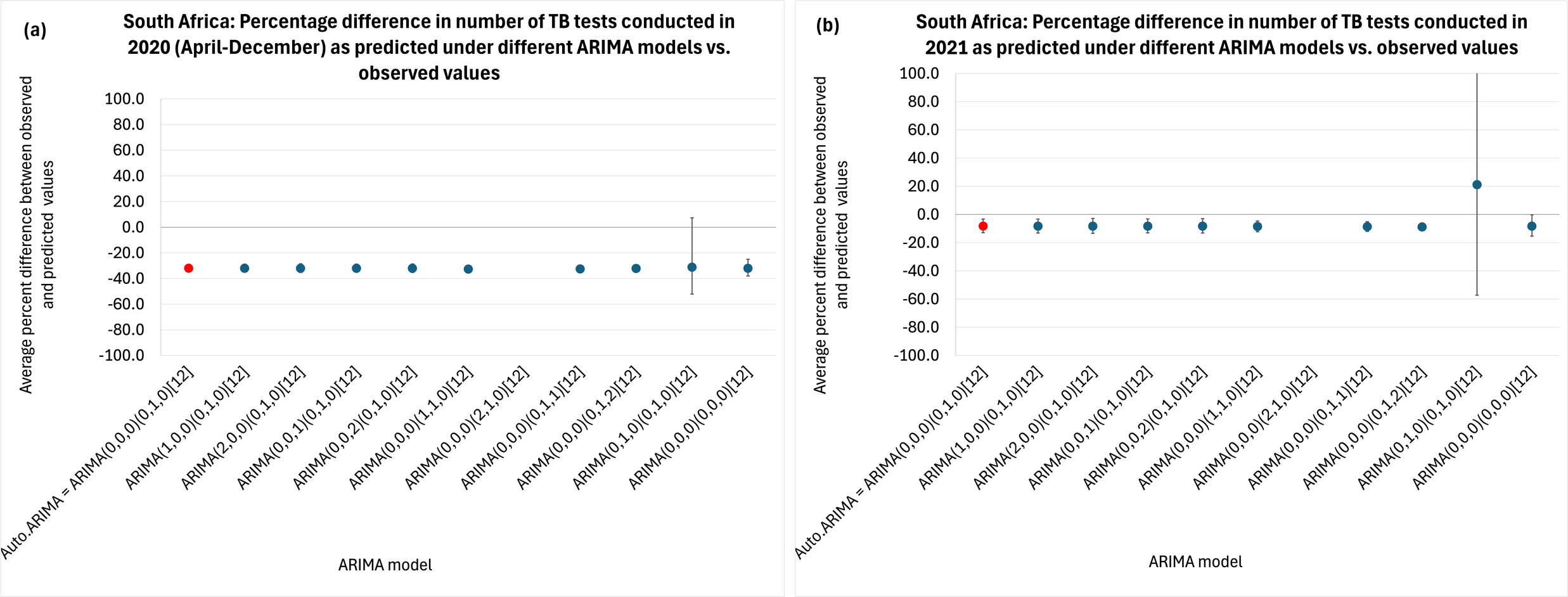

Supplement: S5 Fig — Panel (a) shows percentage differences for 2020 (April–December), and panel (b) for 2021. The Auto.ARIMA model used in the primary analysis is highlighted in red. Error bars represent uncertainty intervals for each model’s prediction. (TIF) [file pgph.0003309.s012.tif]
